# ST elevation in lead aVR – a marker of critical left main coronary artery stenosis: A series of case reports and literature review

**DOI:** 10.1097/MD.0000000000051001

**Published:** 2026-10-09

**Authors:** May Thu Kyaw

**Affiliations:** a University of Medicine 2, Yangon, Myanmar; b Heart and Vascular, Victoria Hospital, Yangon, Myanmar.

**Keywords:** case reports, lead aVR, left main coronary artery stenosis, ST-segment elevation

## Abstract

**Rationale:**

Lead augmented vector right (aVR) has historically been overlooked in acute coronary syndromes. ST-segment elevation (STE) in lead aVR with diffuse ST depression in multiple leads has emerged as a critical marker for left main coronary artery (LMCA) stenosis and triple-vessel disease (3VD). Recognition of this high-risk electrocardiogram (ECG) pattern is essential for timely diagnosis and improved outcomes.

**Patient concerns:**

In this retrospective case series, 5 consecutive patients presented to the emergency department between January 2023 and June 2024 with acute chest pain or cardiac arrest.

**Diagnoses:**

All 5 patients exhibited ECG findings of STE in aVR (≥1 mm) with diffuse ST depressions (≥1 mm) in ≥6 leads. All underwent coronary angiography within 24 hours, which confirmed LMCA stenosis (70%–99%; mean STE in aVR: 3.0 mm). A narrative literature review was performed using PubMed/MEDLINE databases (2000–2024) with search terms including “lead aVR,” “ST-segment elevation,” “left main coronary artery,” and “acute coronary syndrome.” Literature review shows that STE ≥0.5 mm in aVR with diffuse ST depression has been reported to have a sensitivity of 78% and specificity of 86% for LMCA/triple-vessel disease in prior studies. However, it is important to recognize that this ECG pattern can occur in other conditions, including severe anemia, shock states, and aortic dissection. STE ≥1 mm increases diagnostic yield. Patients with this pattern have higher in-hospital mortality (8.6%–19.4% vs 1.3%) and increased heart failure and reinfarction.

**Interventions:**

A total of 2 patients underwent coronary artery bypass grafting, 2 underwent percutaneous coronary intervention, and 1 was managed medically.

**Outcomes:**

No in-hospital deaths occurred.

**Lessons:**

STE in aVR with diffuse ST depression represents a high-risk ECG pattern that should prompt urgent invasive evaluation. Recognition of this high-risk ECG pattern is essential for timely diagnosis of critical LMCA stenosis and improved outcomes. However, findings from this small case series require validation in larger prospective cohorts.

## Graphical abstract

**Figure:**
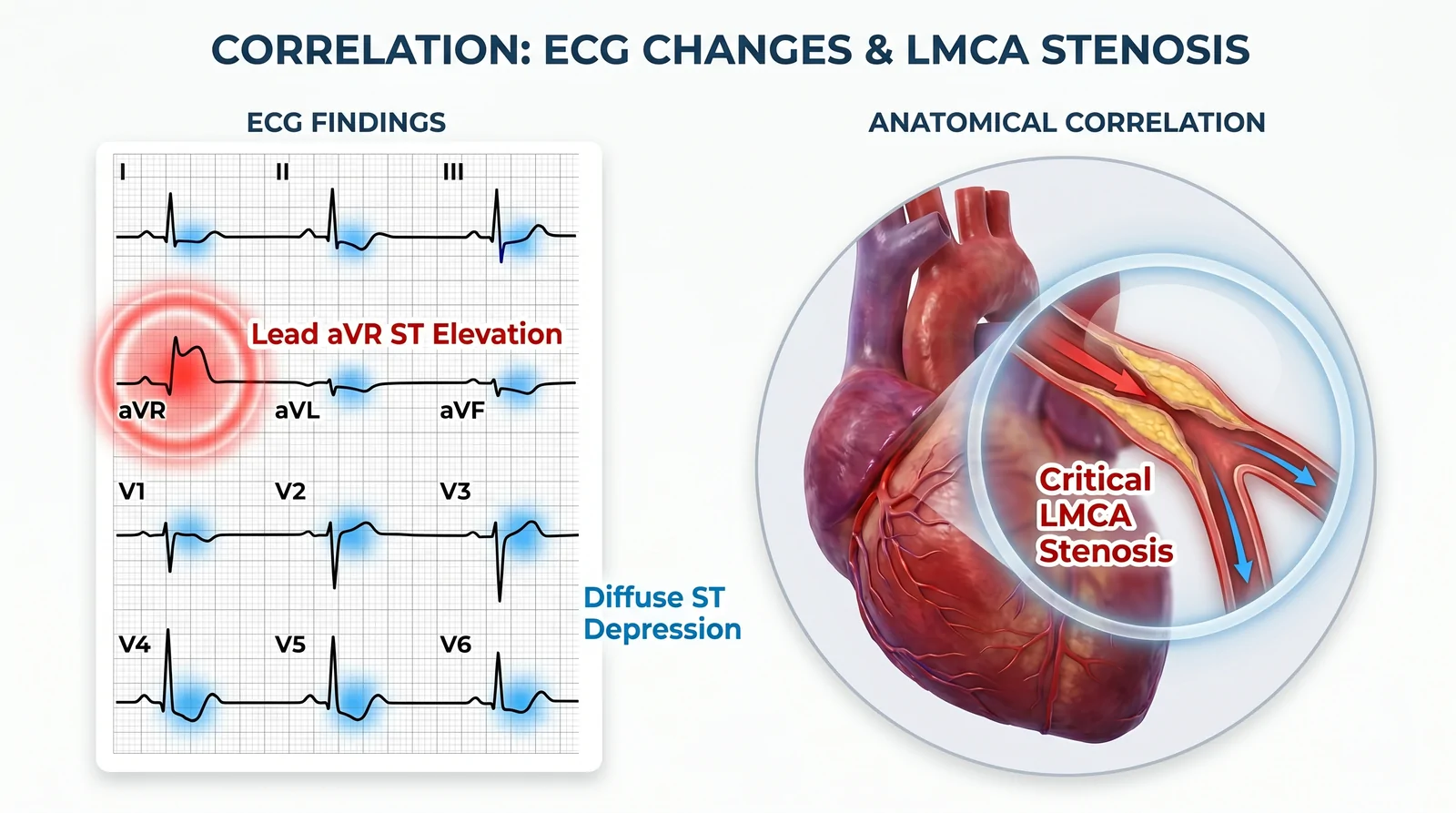

## 1. Introduction

Lead augmented vector right (aVR) has historically been overlooked in the interpretation of electrocardiograms (ECGs) during acute coronary syndromes (ACS), often dismissed as reciprocal to changes in other leads. However, accumulating evidence has established that ST‑segment elevation (STE) in lead aVR, particularly when accompanied by diffuse ST depressions in multiple leads, represents a critical finding with significant diagnostic and prognostic implications.^[1]^ The fourth Universal Definition of Myocardial Infarction has appropriately recognized STE in lead aVR with specific repolarization patterns as a high-risk ECG pattern warranting urgent attention.^[2]^ A left main stem stenosis is defined as a lesion occupying ≥50% of the vessel lumen, though lesions ≥70% are typically considered hemodynamically significant and are the focus of our case reports. Despite this recognition, lead aVR changes remain widely ignored in clinical practice, with chest pain patients exhibiting this pattern traditionally classified as having high‑risk non‑ST elevation ACS rather than being recognized as requiring emergent reperfusion therapy.^[3]^

The ECG abnormality of concern is characterized by prominent STE in lead aVR – often reaching 5 mm or more – accompanied by diffuse ST‑segment depressions (STDs) in 6 or more leads. This pattern reflects widespread subendocardial ischemia and has been strongly associated with left main coronary artery (LMCA) obstruction, proximal left anterior descending artery (LAD) occlusion, and triple-vascular disease (3VD).^[4]^ Importantly, STE in lead aVR is not pathognomonic for LMCA stenosis. Differential diagnoses include severe anemia, drug overdose‑induced vasospasm and tachycardia, aortic dissection involving the LMCA, acute pulmonary embolism, acute pericarditis, critical aortic stenosis, Brugada syndrome, tricyclic antidepressant toxicity, and Takotsubo syndrome. Knotts et al^[5]^ reported that only approximately one‑third of patients presenting with STE in aVR had ACS as the underlying etiology, with tachyarrhythmias and noncardiac shocks accounting for the remainder.

In this manuscript, we present 5 case reports of patients presenting with STE in aVR and diffuse ST depressions who were subsequently found to have critical LMCA stenosis, and we provide a comprehensive narrative literature review synthesizing current evidence regarding the diagnostic accuracy, pathophysiological mechanisms, and prognostic significance of this under-recognized ECG pattern.

## 2. Methods

### 2.1. Study design and patient selection

We conducted a retrospective case series of consecutive patients presenting to our emergency department between January 2023 and June 2024. Patients were identified through review of emergency department records and cardiology consultation logs. Inclusion criteria were: presentation with acute chest pain or cardiac arrest with subsequent return of spontaneous circulation; ECG showing STE ≥ 1 mm in lead aVR; diffuse STD ≥ 1 mm in ≥ 6 leads; and coronary angiography performed within 24 hours of presentation. No exclusion criteria were applied. A total of 5 consecutive patients met these criteria during the study period.

### 2.2. Data collection

Clinical data were extracted from electronic medical records, including demographics, cardiovascular risk factors, presenting symptoms, vital signs, laboratory values (high-sensitivity troponin I), echocardiographic findings, and angiographic results. Coronary angiograms were analyzed using quantitative coronary angiography to determine percentage diameter stenosis.

### 2.3. Literature review

A narrative literature review was performed using PubMed/MEDLINE databases for publications from January 2000 to December 2024. Search terms included “lead aVR,” “ST-segment elevation,” “left main coronary artery,” “acute coronary syndrome,” and combinations thereof. We included original research articles, systematic reviews, meta-analyses, and clinical guidelines in English. Studies were included if they reported on the diagnostic accuracy, prognostic significance, or pathophysiological mechanisms of STE in lead aVR in the setting of ACS. Reference lists of retrieved articles were also screened for additional relevant publications.

### 2.4. Ethical considerations

This study was conducted in accordance with the Declaration of Helsinki. Ethical approval was waived by our institutional review board given the retrospective nature of the study and the use of anonymized clinical data. Written informed consent was obtained from all patients for publication of this case series and any accompanying images.

### 2.5. Case reports

We identified 5 consecutive patients presenting to our emergency department between January 2023 and June 2024 with acute chest pain or cardiac arrest and an ECG showing STE ≥ 1 mm in lead aVR accompanied by diffuse STD (≥1 mm) in ≥6 leads. All 5 underwent coronary angiography within 24 hours, and each had significant LMCA stenosis (≥70% diameter reduction). Their clinical, electrocardiographic, and angiographic characteristics are summarized in Table 1. Representative ECGs and angiograms for cases 1, 2, and 5 are shown in Figures 1A, B, 2A, B, and 3A, B, respectively.

**Table 1:** Clinical, electrocardiographic, and angiographic characteristics of 5 patients with STE in aVR and confirmed LMCA stenosis

Case	Age/sex	Risk factors	STE aVR (mm)	STE V1 (mm)	Leads with STD (n)	LMCA stenosis (%)	Other vessels	Treatment	Peak troponin (ng/mL)	LVEF (%)
1	65/F	HTN, DLP	2	2	6	80 (distal, bifurcation	LAD, LCx	CABG	0.45	
2	72/F	DM, smoking	4	2	7	95 (body)	None	PCI (DES)	28.6	50
3	58/M	CKD, PVD	2.5	1	5	80	3VD	CABG	1.2	
4	48/M	None	5	2	9	99 (Thrombotic occlusion)	None	Thrombectomy + DES	>100	35
5	69/F	HTN, DM	1.5	0	6	70 (Distal)	None	Medical	0.32	

Abbreviations: 3VD = triple-vascular disease, aVR = augmented vector right (also commonly written as augmented voltage right), CABG = coronary artery bypass grafting, CKD = chronic kidney disease, DES = drug‑eluting stent, DLP = dyslipidemia, DM = diabetes mellitus, HTN = hypertension, LAD = left anterior descending, LCx = left circumflex, LMCA = left main coronary artery, LVEF = left ventricular ejection fraction, PCI = percutaneous coronary intervention, PVD = peripheral vascular disease, STD = ST‑segment depression, STE = ST‑segment elevation.

**Figure 1.**
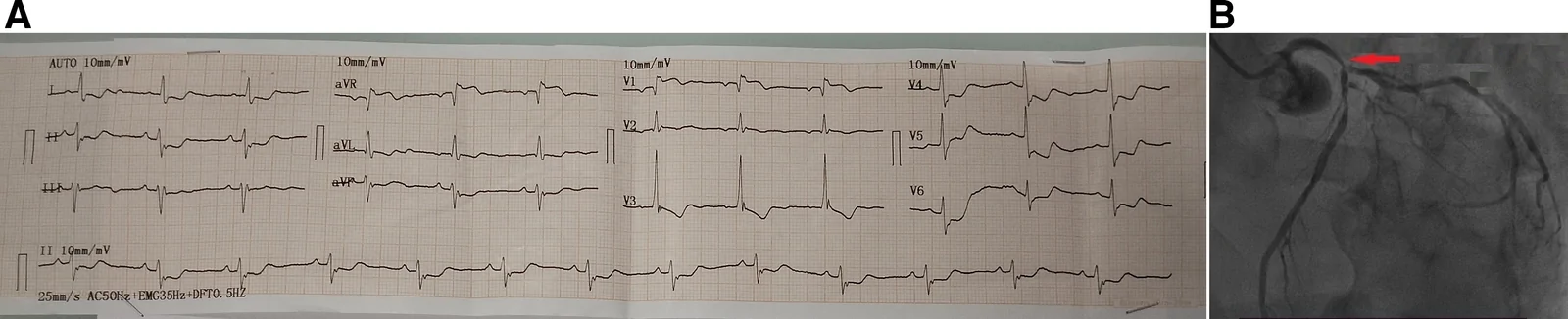
(A) ECG: 2 mm STE in aVR and V1 with 2 to 3 mm STD in I, II, aVF, V4 to V6. (B) Angiography: 80% distal left main stenosis involving LAD and LCx ostia (Medina 1.1.1). aVR = augmented vector right (also commonly written as augmented voltage right), ECG = electrocardiogram, LAD = left anterior descending artery, LCx = left circumflex artery, STE = ST-segment elevation, STD = ST-segment depression.

### 2.6. Representative cases

Case 1 – A 65‑year‑old woman with hypertension and dyslipidemia presented with substernal chest pain radiating to the left arm, lasting 30 minutes. Vital signs were blood pressure 105/70 mm Hg, heart rate 75 bpm. The initial ECG demonstrated 2 mm of STE in leads aVR and V1 with 2 to 3 mm ST depressions in leads I, II, aVF, and V4 to V6 (Fig. 1A). High‑sensitivity troponin I was 0.45 ng/mL (normal < 0.04). Emergency coronary angiography demonstrated 80% tubular stenosis of the distal LMCA extending into the ostioproximal left anterior descending artery (LAD) and left circumflex artery (LCx) (true bifurcation lesion, Medina 1.1.1) (Fig. 1B). The patient underwent successful coronary artery bypass grafting (CABG) with left internal mammary artery to the LAD and saphenous vein grafts to the circumflex and right coronary arteries. Recovery was uneventful.

Case 2 – A 72‑year‑old woman with diabetes mellitus and prior smoking presented with progressive chest tightness over 2 hours. She was hypotensive (90/60 mm Hg) and tachycardic (100 bpm). ECG showed STE of 4 mm in aVR and 2 mm in V1, with diffuse ST depression in leads I, II, aVF, and V3 to V6 (Fig. 2A). Bedside echocardiography revealed severe hypokinesis of the basal septum and anterolateral wall. Emergent coronary angiography revealed 95% stenosis at the body of the LMCA with thrombolysis in myocardial infarction, grade 2 flow (Fig. 2B). Percutaneous coronary intervention with a drug‑eluting stent to the left main was performed successfully. Peak troponin I was 28.6 ng/mL. Left ventricular ejection fraction was 50% at discharge.

**Figure 2.**
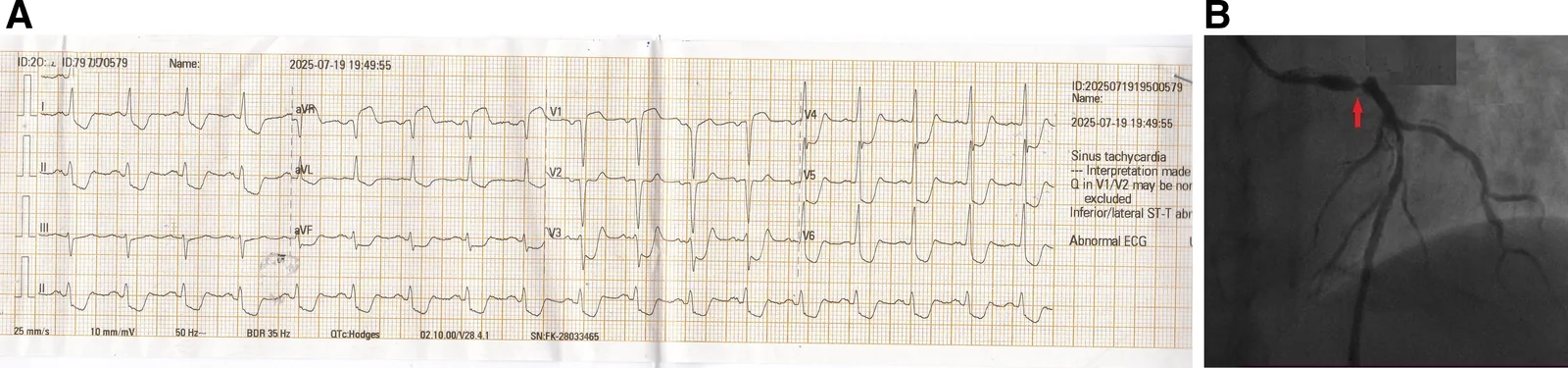
(A) ECG: 4 mm STE in aVR and 2 mm STE in V1 with diffuse STD. (B) Angiography: 95% left main body stenosis (TIMI‑2 flow). TIMI 2: Partial reperfusion (contrast material opacifies the coronary bed distal to the obstruction; however, the rate of entry or clearance of contrast is slower than normal). aVR = augmented vector right (also commonly written as augmented voltage right), ECG = electrocardiogram, STE = ST-segment elevation, STD = ST-segment depression, TIMI-2 = thrombolysis in myocardial infarction, grade 2.

Case 3 – A 58‑year‑old man with chronic kidney disease and peripheral vascular disease presented with recurrent chest pain at rest. ECG on admission showed 2.5 mm STE in aVR and 1 mm STE in V1, with ST depression in leads I, aVL, and V4 to V6. High‑sensitivity troponin I was 1.2 ng/mL. Angiography revealed 80% left main stenosis with diffuse 3‑vessel disease (Syntax score 28). After multidisciplinary discussion, the patient underwent successful CABG. The postoperative course was uneventful.

Case 4 – A 48‑year‑old man with no significant cardiac history presented with out‑of‑hospital cardiac arrest with return of spontaneous circulation after 8 minutes. Although the patient did not report chest pain before arrest, this presentation was considered to represent an ACS equivalent. Postresuscitation ECG demonstrated 5 mm STE in lead aVR, 2 mm STE in V1, and profound ST depression in all other leads except aVL. Emergent angiography revealed 99% thrombotic occlusion of the LMCA. Intravascular ultrasound confirmed heavy plaque burden with superimposed thrombus. Thrombectomy and drug‑eluting stent implantation restored thrombolysis in myocardial infarction, grade 3 flow. Peak troponin was >100 ng/mL, and left ventricular ejection fraction was 35% at discharge.

Case 5 – A 69‑year‑old woman with hypertension and uncontrolled diabetes mellitus presented with intermittent chest pain for 12 hours. ECG showed 1.5 mm STE in aVR without V1 involvement, and ST depression in leads II, aVF, and V3 to V6 (Fig. 3A). High‑sensitivity troponin I was 0.32 ng/mL. Angiography revealed distal left main 70% stenosis with no other significant lesions (Fig. 3B). Given the absence of ongoing ischemia, normal myocardial perfusion on stress testing using myocardial perfusion scintigraphy (MPS) with technetium-99m sestamibi, which demonstrated no significant perfusion defects at rest or with pharmacological stress, and stable hemodynamics, the patient was treated conservatively after multidisciplinary discussion. Medical management included high-intensity statin and dual antiplatelet therapy. The patient’s asymptomatic status, despite uncontrolled diabetes, was attributed to possible diabetic neuropathy rather than absence of ischemia, though no formal autonomic testing was performed. Repeat angiography at 6 months showed stable disease, and the patient remained symptom‑free.

**Figure 3.**
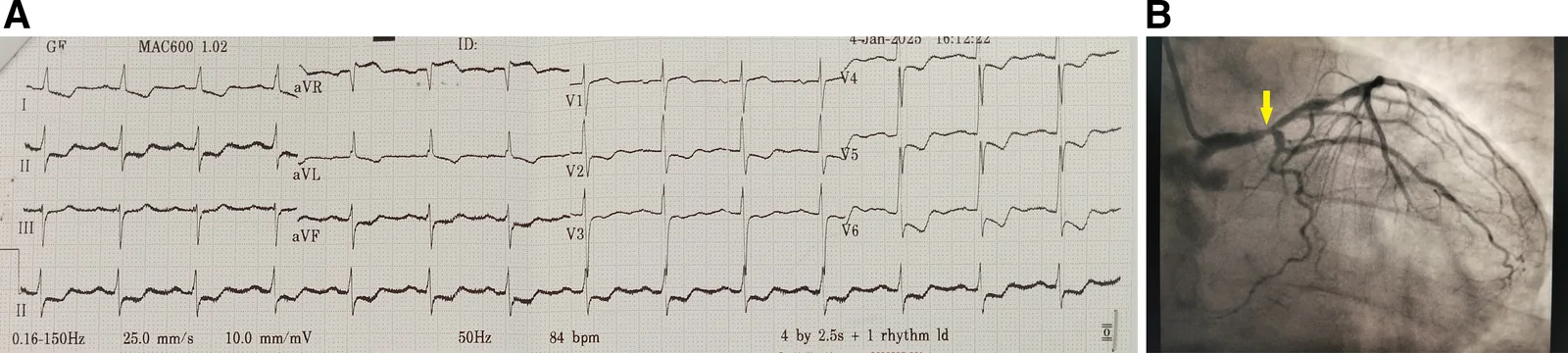
(A) ECG: 1.5 mm STE in aVR (V1 negative) with STD in II, aVF, V3 to V6. (B) Angiography: 70% distal left main stenosis without other lesions. aVR = augmented vector right (also commonly written as augmented voltage right), ECG = electrocardiogram, STE = ST-segment elevation, STD = ST-segment depression.

Summary: All 5 patients had angiographically significant LMCA stenosis (range: 70%–99%). Mean STE in lead aVR was 3.0 mm (range: 1.5–5 mm). Two patients underwent CABG (cases 1 and 3), 2 underwent percutaneous coronary intervention (cases 2 and 4), and 1 was managed medically (case 5). There were no in‑hospital deaths.

## 3. Discussion

### 3.1. Diagnostic utility of STE in lead aVR

The diagnostic utility of STE in lead aVR for detecting LMCA disease has been extensively validated in the published literature. In prior studies, STE in lead aVR was present in 90.2% of LMCA patients, 34.7% of those with 3‑vessel disease, 10.5% of LAD occlusions, and 6.3% of right coronary artery occlusions. Notably, no patients with isolated LCx occlusion exhibited this finding.^[6]^ Kosuge et al ^[7]^ reported a sensitivity of 78% and specificity of 86% for STE > 0.5 mm in lead aVR in predicting left main or 3‑vessel disease.

The magnitude of ST elevation correlates directly with diagnostic accuracy and disease severity. STE ≥ 1 mm increases sensitivity for LMCA disease to 35% and for 3VD to 60.4%, while STE ≥ 1.5 mm achieves 98% specificity for LM/3VD.^[8,9]^ The combination of STE in aVR with diffuse ST depressions in 8 or more leads demonstrates a predictive accuracy of 75% for LMCA or TVD.^[10]^ When STE in aVR is accompanied by STE in lead V1, specificity for LMCA stenosis increases to 68.5%, though sensitivity decreases to 74.4%.^[11]^

Importantly, while these diagnostic parameters demonstrate the utility of this ECG pattern, they must be interpreted with caution. This ECG pattern can occur in non-ACS conditions, including severe anemia, hemorrhagic shock, septic shock, tachyarrhythmias, and aortic dissection. Therefore, the findings should be considered in the appropriate clinical context rather than as definitive diagnostic criteria in isolation.

### 3.2. Pathophysiological mechanisms

From a pathophysiological perspective, lead aVR reflects electrical activity from the right ventricular outflow tract and basal interventricular septum – regions typically perfused by proximal septal branches of the LAD.^[12]^ STE in lead aVR may result from 2 mechanisms: transmural ischemia of the basal septum, or reciprocal changes from diffuse subendocardial ischemia affecting the lateral and inferior walls. In LMCA obstruction, posterior wall ischemia from circumflex compromise counterbalances anterior wall ischemic forces. This produces more prominent STE in aVR than in precordial leads, distinguishing this pattern from isolated LAD occlusion, where STE in V1 typically exceeds that in aVR.^[10,13]^

An important distinction exists between total and subtotal LMCA occlusion. Subtotal occlusion typically produces the pattern of diffuse ST depression with STE in aVR and often V1, reflecting circumferential subendocardial ischemia.^[14]^ Total LMCA occlusion without collateral circulation may present as a ST-elevation myocardial infarction (STEMI) pattern with STE in leads I, aVL, and V2 to V6. Patients with well‑developed collateral circulation and total occlusion may paradoxically present with the subtotal pattern.^[15]^ Recognition of this distinction is clinically relevant, as the non-ST-elevation myocardial infarction pattern of diffuse ST depression with STE aVR warrants the same urgency as STEMI.

### 3.3. Prognostic significance

The prognostic significance of STE in aVR is substantial. Barrabés and colleagues demonstrated that STE of 0.05 to 0.1 mV in lead aVR increased in‑hospital death risk over fourfold, while STE ≥ 0.1 mV increased risk nearly 7-fold, with corresponding mortality rates of 8.6% and 19.4% compared with 1.3% in patients without STE aVR.^[16]^ STE in aVR has been independently associated with higher 30‑day mortality in both anterior and inferior myocardial infarction, with odds ratios of 1.69 for anterior MI with STE ≥ 1.5 mm and 2.41 for inferior MI with STE ≥ 1 mm.^[17]^

### 3.4. Clinical guidelines and management

The 2020 European Society of Cardiology Guidelines for non‑ST elevation-ACS classify patients with ST depression ≥ 1 mm in ≥6 leads with simultaneous STE in leads aVR and/or V1 as very high‑risk, recommending an immediate invasive strategy within 2 hours.^[18]^ Similarly, the 2013 American College of Cardiology Foundation/American Heart Association STEMI guidelines added multilead ST depression with coexistent STE in aVR as an indication for acute reperfusion therapy.^[19]^ The American Heart Association recommends that automated ECG interpretation of ST depression >0.1 mV in 8 or more leads with STE in aVR and/or V1 should suggest “ischemia because of multivessel or LMCA obstruction.”^[20]^

While these guidelines appropriately emphasize the urgency of this ECG pattern, it is important to recognize that STE in aVR with diffuse ST depression should be considered a high-risk pattern rather than directly equated with STEMI, as the underlying pathophysiology and management considerations may differ.

### 3.5. Differential diagnoses

Clinicians must remain aware of non‑ACS etiologies of STE in aVR, including severe anemia, hemorrhagic shock, septic shock, tachyarrhythmias, and aortic dissection. A thorough history and physical examination, along with appropriate laboratory and imaging studies, are essential before initiating anticoagulation or antiplatelet therapy, particularly when surgical revascularization may be required.

### 3.6. Limitations

Several limitations of this study warrant acknowledgments. First, the sample size is quite small (n = 5), reflecting the relatively low prevalence of LMCA lesions and the fact that many patients with such lesions may not survive to undergo angiography because of arrhythmic complications. Although our results are consistent with the broader literature, the small cohort limits the generalizability of our findings. Second, the retrospective design and absence of a comparison group (e.g., patients with STE in aVR but without LMCA disease) precludes calculation of specificity or predictive values from our case series. Third, our literature review was narrative rather than systematic, lacking a formalized search strategy with explicit inclusion/exclusion criteria and quality assessment of included studies. Fourth, in case 5, while the patient was treated medically, the absence of systematic ischemia testing beyond the MPS performed – which demonstrated no significant perfusion defects – represents a limitation. The MPS was performed using technetium-99m sestamibi with pharmacological stress, and while the results supported conservative management, invasive fractional flow reserve measurement was not performed to definitively assess the hemodynamic significance of the 70% stenosis. The patient’s asymptomatic status despite uncontrolled diabetes raises the possibility of diabetic neuropathy masking ischemic symptoms, though this was not formally evaluated. Fifth, we did not systematically assess for non-ACS etiologies of STE in aVR in our cohort, though clinical evaluation did not suggest alternative diagnoses. Sixth, the inclusion of a cardiac arrest patient (case 4) in a case series with an inclusion criterion of chest pain represents an inconsistency in our methodology that we have addressed by expanding the inclusion criteria. Future prospective studies with larger sample sizes and control groups are needed to validate these findings.

## 4. Conclusion

STE in lead aVR with diffuse ST depression in multiple leads is a high‑risk ECG pattern that mandates urgent recognition and intervention. This finding demonstrates strong diagnostic accuracy for critical LMCA stenosis and TVD in the published literature, with sensitivity approaching 100% for LMCA lesions at lower thresholds and specificity exceeding 95% at higher thresholds. The prognostic implications are substantial, with STE in aVR independently predicting in‑hospital mortality, heart failure, and recurrent ischemic events.

Current international guidelines appropriately classify this ECG pattern as a high-risk finding warranting immediate invasive coronary angiography within 2 hours of presentation. Clinicians must maintain a broad differential diagnosis, as non‑ACS conditions including severe anemia, shock states, and aortic dissection can produce identical ECG findings. While this pattern should not be directly equated with STEMI in all cases, in the appropriate clinical context of suspected ACS, STE in aVR with diffuse ST depression should trigger prompt catheterization laboratory activation. Recognition of this long‑overlooked but highly valuable lead will improve timely diagnosis of life‑threatening coronary anatomy and ultimately enhance patient outcomes. However, larger prospective studies are needed to further validate these findings.

## Author contributions

**Conceptualization:** May Thu Kyaw.

**Investigation:** May Thu Kyaw.

**Resources:** May Thu Kyaw.

**Supervision:** May Thu Kyaw.

**Writing** – **original draft:** May Thu Kyaw.

**Writing** – **review & editing:** May Thu Kyaw
